# Urinary metabolic profiling by ^1^H NMR spectroscopy in patients with cirrhosis may discriminate overt but not covert hepatic encephalopathy

**DOI:** 10.1007/s11011-016-9904-0

**Published:** 2016-09-17

**Authors:** Mark J. W. McPhail, Sara Montagnese, Manuela Villanova, Hamza El Hadi, Piero Amodio, Mary M. E. Crossey, Roger Williams, I. Jane Cox, Simon D. Taylor-Robinson

**Affiliations:** 1 Liver Unit, Division of Digestive Health, Department of Surgery and Cancer, Imperial College London, St Mary’s Campus, London, W2 1NY UK; 2 Department of Medicine DIMED, University of Padova, Padova, Italy; 3 Institute of Hepatology London, Foundation for Liver Research, 111 Coldharbour Lane, London, SE5 9NT UK; 4 Faculty of Life Sciences & Medicine, King’s College London, London, UK

**Keywords:** Hepatic encephalopathy, Metabolic profiling, Urinary biomarkers, Magnetic resonance spectroscopy, Hippurate, Histidine

## Abstract

To date urinary metabolic profiling has been applied to define a specific metabolic fingerprint of hepatocellular carcinoma on a background of cirrhosis. Its utility for the stratification of other complications of cirrhosis, such as hepatic encephalopathy (HE), remains to be established. Urinary proton nuclear magnetic resonance (^1^H-NMR) spectra were acquired and NMR data from 52 patients with cirrhosis (35 male; 17 female, median (range) age [60 (18–81) years]) and 17 controls were compared. A sub-set of 45 patients (33 male; 12 female, [60 (18–90) years, median model for end stage liver disease (MELD) score 11 (7–27)]) were fully characterised by West-Haven criteria, Psychometric Hepatic Encephalopathy Score (PHES) and electroencephalogram (EEG), and defined as overt HE (OHE, *n* = 21), covert HE (cHE, *n* = 7) or no HE (*n* = 17). Urinary proton nuclear magnetic resonance (^1^H-NMR) spectra were analysed by partial-least-squares discriminant analysis (PLS-DA). The results showed good discrimination between patients with cirrhosis (*n* = 52) and healthy controls (*n* = 17) (R2X = 0.66, R2Y = 0.47, Q2Y = 0.31, sensitivity-60 %, specificity-100 %) as the cirrhosis group had higher 1-methylnicotinamide with lower hippurate, acetate, phenylacetylglycine and N-methyl nicotinic acid levels. While patients with OHE could be discriminated from those with no HE, with higher histidine, citrate and creatinine levels, the best models lack robust validity (R2X = 0.65, R2Y = 0.48, Q2Y = 0.12, sensitivity-100 %, specificity-64 %) with the sample size used. Urinary ^1^H-NMR metabolic profiling did not discriminate patients with cHE from those without HE, nor discriminate subjects on the basis of PHES/EEG result or MELD score. In conclusion, patients with cirrhosis showed different urinary ^1^H-NMR metabolic profiles compared to healthy controls and those with OHE may be distinguished from those with no HE although larger studies are required. However, urinary ^1^H-NMR metabolic profiling did not discriminate patients with differing grades of HE or according to severity of underlying liver disease.

## Introduction

Hepatic encephalopathy (HE) is a common complication of cirrhosis, porto-systemic shunting and acute liver failure (ALF) (Ferenci et al. 2002;Vilstrup et al. 2014). HE is a defining feature of ALF and the majority of patients with cirrhosis will experience an episode of HE at some point during their illness. This may manifest as confusion, somnolence, poor concentration or even coma. In some patients such overt symptoms are lacking, but when psychometric tests are performed, significant impairment is revealed in attention, concentration and executive function (Weissenborn et al. 2001;Weissenborn 2013). Such covert HE (cHE) is associated with progression to OHE with hospitalisation and has been linked to shortened survival in some studies (Weissenborn 2015).

The pathogenesis of HE is incompletely understood. Hyperammonaemia is central to HE, but ammonia levels only correlate with the cerebral oedema and outcome of patients with ALF. In ALF, gut-derived ammonia is not detoxified in the liver, due to failure of the urea cycle, causing large rises in ambient ammonia levels. Following translocation across the blood brain barrier, ammonia enters astrocytes and is converted to glutamine. Whether by glutamine-associated osmosis or failure of mitochondria (due to ammonia toxicity), the astrocyte swells and symptoms related to cerebral oedema occur.

However, although present in patients with HE neither hyperammonaemia nor cerebral oedema correlate strongly with severity of HE in patients with cirrhosis and co-factors are required to explain the clinical syndrome and brain dysfunction. These include the presence of inflammatory states, such as sepsis (Tranah et al. 2013). Furthermore, the role of the gut microbiota have recently been implicated in HE (Bajaj et al. 2013), which have been explored in proof-of-principle studies, assessing the effect of therapies, such as lactulose (Bajaj et al. 2012) and rifaximin (Bajaj et al. 2011).

The role of zinc (Warthon-Medina et al. 2015) and manganese (Rivera-Mancia et al. 2011) have long been reported in patients with cognitive dysfunction and liver disease. Low zinc levels are common in acute and chronic liver disease and are associated with increased GABAergic tone. Manganese is deposited in the basal ganglia in patients with cholestasis (including when secondary to parenteral nutrition) and in cirrhosis and may contribute to the Parkinsonian phenotype observed in chronic encephalopathy (Zeron et al. 2011).

Hyponatraemia is a common cofactor in cerebral oedema in patients with acute liver failure and traumatic brain injury where hypertonic saline solutions are indicated as prophylaxis or treatment for raised intracranial pressure. In cirrhosis patients with hyponatraemia from diuretic use, dilution and hepatorenal syndrome are also at increased risk of overt HE (Iwasa and Takei 2015).

Present diagnostic pathways for HE involve bedside clinical assessment, psychometric evaluation (pencil-and-paper or computerized test batteries), and electroencephalography (EEG). While structural and functional magnetic resonance imaging can elucidate profound changes associated with HE, these powerful imaging modalities are not yet suitable for routine clinical diagnostic use. A “biomarker” for grading HE would be of significant importance to the hepatology community. At present, plasma or capillary ammonia remains the only plasma marker in routine use. This measurement has significant false positive rates (although virtually no false negative rates), because of the multifactorial nature of HE and relative instability of ammonia outside the body at ambient temperatures. Recent evidence suggests that the gut microbiota can be interrogated by salivary microbe analysis, which reflects gut host-microbe interactions and inflammation, another important cofactor in the development of HE (Bajaj et al. 2015).

Metabolic profiling (Nicholson et al. 2012) (also termed metabonomics or metabotyping) involves analysis of biofluids or tissues by measurement of low molecular weight (<1 kDa) compounds using proton nuclear magnetic resonance (^1^H-NMR) spectroscopy or mass spectrometry (MS) techniques (Nicholson et al. 1999). Alterations in the complex spectral data sets can then be assessed using multivariate statistical techniques (Trygg et al. 2007) to determine scope of the data sets for diagnosis, prognosis or response to intervention. Since the metabolic profile is typically comprised of hundreds or thousands of signals, depending on the technique, it might prove to be a highly valuable methodology in delivering personalised and highly discriminant prediction of response or diagnostic accuracy (Nicholson and Holmes 2006).

It has recently been shown that information about the gut-liver-brain axis can be determined from urinary ^1^H-NMR analysis (Williams et al. 2008;Williams et al. 2010). Urinary metabolic profiles have been noted to be useful in classifying patients with non-HE related complications of cirrhosis, such as hepatocellular carcinoma (Ladep et al. 2014;Shariff et al. 2010;Shariff et al. 2011), but the relative contribution from HE to the urinary metabolome remains under-characterised.

A recent proton magnetic resonance spectroscopy of metabolic profiling of serum in patients with HE, showed that supervised modelling provided discrimination between healthy controls and patients with cirrhosis (Jimenez et al. 2010). A predictive model was generated which displayed strong discrimination between patients with and without cHE. cHE patients displayed increased serum concentrations of glucose, lactate, methionine, trimethylamine-*N*-oxide (TMAO), and glycerol, as well as decreased levels of choline, branch amino acids, alanine, glycine, acetoacetate, NAC, and lipid moieties. Defining metabolic change in urine samples of patients with cHE would strengthen the validity of metabolic profiling as a future diagnostic and research tool in patients with HE.

In this study, the urinary ^1^H-NMR metabolic profile from a well characterised group of patients with cirrhosis, in the presence and absence of HE, was interrogated with clinical and diagnostic assessments to determine the utility of urinary metabolic phenotyping for assessing cHE.

## Materials and methods

This study conformed to the ethical standards of the Declaration of Helsinki and underwent review and approval at Hammersmith and Queen Charlottes & Chelsea Local Research Ethics Committee (ref 04/Q0406/161) and Padova University Hospital Ethical Review Board (Protocollo 1385P, and subsequent 2010 amendments).

## Patient selection

**Fig. 1:**
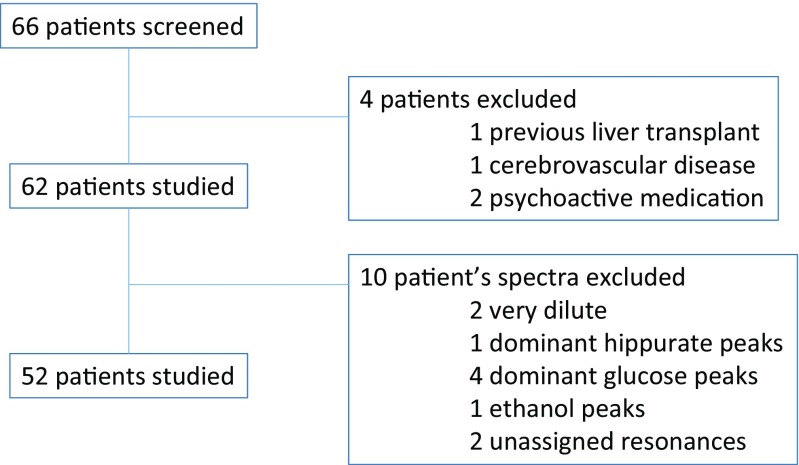
Flowchart of patient recruitement

Sixty-six consecutive patients with cirrhosis referred to the outpatient clinic for Cognitive Disturbances in Medicine, Padova University Hospital. Patients were excluded (4 out of 66 screened) if they were actively misusing alcohol, had significant cerebrovascular or cardiovascular disease, renal failure, neurological or psychiatric co-morbidity, previous liver transplant, were taking psychoactive drugs or were unable/unwilling to participate (see Fig. 1 for flow chart of recruitment; demographic and liver failure characteristics are presented in Table 1, by degree of neuropsychiatric impairment, vide infra). Twenty age-matched healthy volunteers served as controls.

**Table 1:** Characteristics of included patients for multivariate analysis and with full clinical data

Variable	Patients with cirrhosis	OHE	cHE	No HE	*P*-value
N	45	21	7	17	
Age median (range)	60 (18–90)	60(18–90)	67 (42–81)	43(58–74)	0.368*
Sex (M:F)	33:12	14:7	6:1	13:4	0.152^
MELD median (range)	11 (7–27)	11(7–23)	10(7–12)	8(11–27)	0.176*
Abnormal EEG (Y:N)	23:22	17:4	6:1	0:17	<0.002^
Abnormal PHES (Y:N)	16:29	14:7	1:6	0:17	<0.001^
BMI	25(18–37)	24(18–35)	32 (24–37)	25 (18–34)	0.101*

*Kruskall Wallis test, ^-χ2 test. OHE overt hepatic encephalopathy, cHE covert hepatic encephalopathy. MELD model for end stage liver disease, BMI body mass index

## Neurophysiological assessment

Patients underwent 10-min, eyes-closed EEG recording, in a condition of relaxed wakefulness and in a quiet room, at 08:30–09:00 in the morning. Attention was paid to avoid somnolence, muscle or other types of artefact. The EEG was obtained by Brainquick 3200 digital equipment (Micromed, Italy). A 21-channel cap was used, and the electrodes placed according to the 10–20 International System (ground: Fpz, reference Oz). Impedance was kept under 5 kΩ. Each channel had its own analogue-to-digital converter, while signals were digitally filtered in the 1.6–70 Hz range. Sampling frequency was 256 Hz, with 12-bit analogue-to-digital conversion. One continuous 80–100 s period of EEG tracing was selected (authors SM and/or PA) for subsequent spectral analysis by Fast Fourier Transform. The following spectral parameters were obtained: mean dominant frequency (MDF), which is an estimate of the background frequency of the EEG, and relative power of the spectral bands delta (1–3.5 Hz), theta (4–7.5 Hz), alpha (8–13 Hz) and beta (13.5–26.5). Spectral parameters were obtained on the derivations P3-P4 (bi-parietal), and the EEG was qualified as normal/abnormal, according to Amodio and co-workers (Amodio et al. 1999).

## Neuropsychological assessment

Psychometric performance was assessed, under standardized conditions, using Number Connection Tests A and B, the Digit Symbol, Line Tracing, and Serial Dotting tests (Weissenborn et al. 2001). Results were scored with relation to age and education-adjusted Italian norms (Amodio et al. 2008). Performance was classified as impaired if the sum of the standard deviations for the individual tests, referred to as Psychometric Hepatic Encephalopathy Score (PHES), was ≤ −4 (Amodio et al. 2008).

## Neuropsychiatric status

Neuropsychiatric status on the day of study was classified as *unimpaired*: no clinical evidence of HE and normal PHES; *covert HE:* no clinical abnormalities but abnormal PHES and/or abnormal EEG; *overt HE:* clinically evident neuropsychiatric disturbances [≥ grade II according to the West Haven criteria (Conn 1977), applied by authors SM and/or PA] (Vilstrup et al. 2014).

## Nutritional status

This included measurement of weight/height, calculation of the body mass index (BMI), and evaluation of body composition by the mid-arm circumference and the triceps skinfold thickness. The mid-arm muscular area was then calculated and the results scored according to reference percentiles (Frisancho 1981). In addition, information was obtained to calculate the Royal Free Hospital (RFH) nutritional screening tool and patients qualified as being at high, medium or low risk for malnutrition (Morgan et al. 2006).

No dietary exclusion was imposed on the participating subjects, but a detailed dietary and lifestyle history was taken. This included current regular medications, and any other drugs used intermittently; use of any herbal remedies or medications, including pre- or probiotics; usual and recent alcohol intake; smoking history; exercise and normal dietary habits; and a 24 h dietary recall. Participants were also directly questioned about their intake of specific dietary components which may influence urinary metabolic profiles.

## Urinary metabolic profiling by ^1^H-NMR spectroscopy

Samples were collected from random mid-stream urine between 10.00 and 16.00. The urine samples were centrifuged at 2500 rpm for 20 min to remove precipitates and then stored in siliconized microvials at -80 °C pending subsequent metabolic profiling using nuclear magnetic resonance (NMR) spectroscopy techniques.

Samples were thawed and prepared as follows: 400 μL of urine was mixed with 200 μL of buffer solution (0.2 M Na_2_HPO_4_/0.2 M NaH_2_PO_4_, pH 7.4), and 60 μL of 3-trimethylsilyl-(2,2,3,3-^2^H_4_)-1-propionate (TSP)/D_2_O solution was added. The TSP served as an internal chemical shift reference (δ 0.00 ppm) and the D_2_O provided a field lock. The buffered urine sample was left to stand and then centrifuged at 13,000 *g* for 10 min. A total of 550 μL of supernatant was transferred into a 5 mm diameter glass NMR tube (Norell Inc., Landisville, NJ, USA).

^1^H-NMR spectra were acquired at 25 °C using a pulse-collect sequence with water presaturation (JEOL 500 MHz Eclipse + NMR spectrometer). Sixty-four data collects were summated. A 90° pulse angle was used with an acquisition time of 4.4 s and a total repetition time of 6.4 s. The ^1^H-NMR spectra were pre-processed using the KnowItAll Informatics System v7.8 (Bio-Rad, Philadelphia, PA). Free induction decays were zero-filled by a factor of 2 and multiplied by an exponential window function with a 0.3 Hz line-broadening factor prior to Fourier transformation. All ^1^H-NMR spectra were phased and a baseline correction applied manually. ^1^H-NMR spectral resonances were assigned on the basis of chemical shifts and coupling patterns and according to the literature (Bouatra et al. 2013;Holmes et al. 1997). ^1^H-NMR spectral analysis included the range δ 0.20–10.00 ppm, excluding the region δ 4.50–6.40 ppm, to remove the residual water and urea signal.

## Data analysis

Principal Component Analysis (PCA) was performed to visualise any inherent clustering and identify outliers using the Hotelling’s t ellipse for strong outliers at the 95 % confidence interval. Orthogonal Projection to Latent Structure (OPLS) analysis was performed to supervise class differences while minimising variability unrelated to class. The R^2^ value was calculated to give a measure of the goodness of fit or amount of variability explained by the model. A cross-validated Q^2^ statistic (based on a 1:7 leave one out algorithm) was calculated as a quantitative measure of the predictability of the model for the Y variable, where a positive Q^2^ indicated good predictivity. In 2-class discriminant problems the Q^2^ value may not be the best method for determining predictivity or validity, so a number of other measures were also performed.

Permutation analysis allows assessment of whether over-fitting is occurring (e.g. due to excess number of components to generate high R^2^ and Q^2^). 999 random permutations were calculated using partial least squares discriminant analysis (PLS-DA) models using the same number of components as orthogonal projection on latent structure-discriminant analysis (OPLS-DA). The cross-validated-analysis-of-variance (CV-ANOVA) statistical assessment corresponds to a null-hypothesis of equal predictive residuals between the models under investigation. A value <0.05 rejects the null hypothesis and suggests the model fitted is superior to one chosen at random. Sensitivity and specificity were calculated from the Y predicted variable either back predicting via a leave-one-out algorithm on the cross-validated data set (internal validation) or by modelling on a random 50 % of the data set and predicting the class of the remaining 50 % (external validation). The S-plot loadings (p v p(corr)) was investigated to determine the metabolites contributing to class separation both in terms of effect on the model (f(p)) or in terms of confidence in predicting class differences (f(p(corr)). A leave one out cross validation method was used for the sensitivity and specificity analysis.

The spectral regions corresponding to selected metabolite peaks, as identified by the PCA and OPLS-DA loadings plots, were normalised to the sum of the total spectral integral to account for differences in concentration, and differences in these relative metabolite signal levels were compared between groups using the Student’s t test or one way ANOVA (following logarithmic transformation if necessary) with correction for multiple comparisons by the Tukey-Kramer method. In all cases a *two-sided p*-value of <0.05 was considered significant.

## Results

While 62 patients with cirrhosis and 20 controls were recruited to the study, the initial study cohort was revised to comprise of 52 patients with cirrhosis and 17 controls, as there were limitations in the urinary NMR data sets from 10 patients [(very dilute samples (*n* = 2), dominant hippurate signals (*n* = 1), dominant glucose or glucosamine peaks (*n* = 4), ethanol (n = 1) and additional unassigned resonances (n = 2)] and three controls [very dilute sample (n = 1), baseline artefact (n = 1), dominant hippurate (n = 1)].

Of these 52 patients, 40 were taking lactulose, 32 rifaximin and 14 branch-chain amino acids. The majority of patients (*n* = 35) were taking two or more agents. Twelve patients were not on any anti-encephalopathy medication.

**Fig. 2:**
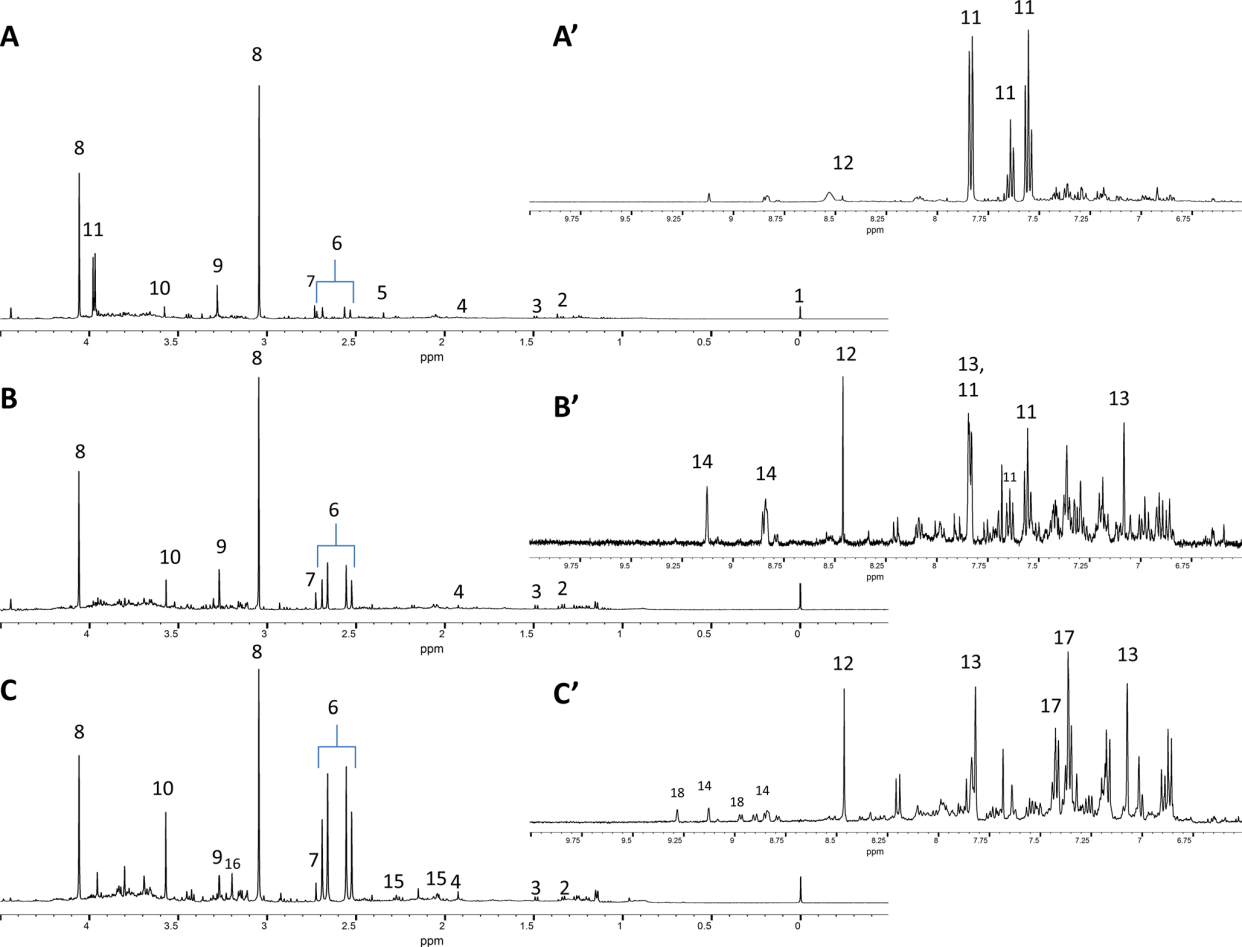
Representative urinary NMR spectra from **A, A'** (hvb10) control; **B, B'** (ptb14) EEG 0, PHES 0, overt 0; and **C**, **C'**: (IP22) EEG, PHES 1, overt 1. Peak assignments 1, added external reference standard (TSP); 2, lactate; 3, alanine; 4, acetate; 5, pyruvate; 6, citrate; 7, dimethylamine; 8, creatinine; 9, trimethylamine-*N*-oxide; 10, glycine; 11, hippurate; 12, formate; 13, histidine; 14, N-methyl nicotinic acid; 15, glutamate; 16, acetylcarnitine; 17, phenylacetylglycine; 18, 1-methylnicotinamide

Not all subjects completed all the clinical assessments and therefore details from the final study cohort of 45 patients (with complete clinical data and urinary NMR profiles) and 17 controls are summarised in Table 1. Twenty-one of these patients had OHE, 7 patients had cHE and 17 patients no HE. Representative urinary ^1^H-NMR spectra from patients with cirrhosis (with and without OHE) and healthy control are presented in Fig. 2A, B and C.

Unsupervised PCA was performed on the initial data set of 69 subjects (17 healthy controls and 52 patients with cirrhosis) to help identify outliers. Clustering occurred for control subjects with significantly increased variation in the urinary NMR spectral from patients with cirrhosis.

A three-component PCA model adequately described the variation in the urinary ^1^H-NMR data sets with acceptable validity (R2X = 0.674, Q2 = 0.54). Principal component 2 separated healthy controls from patients with cirrhosis.

**Fig. 3:**
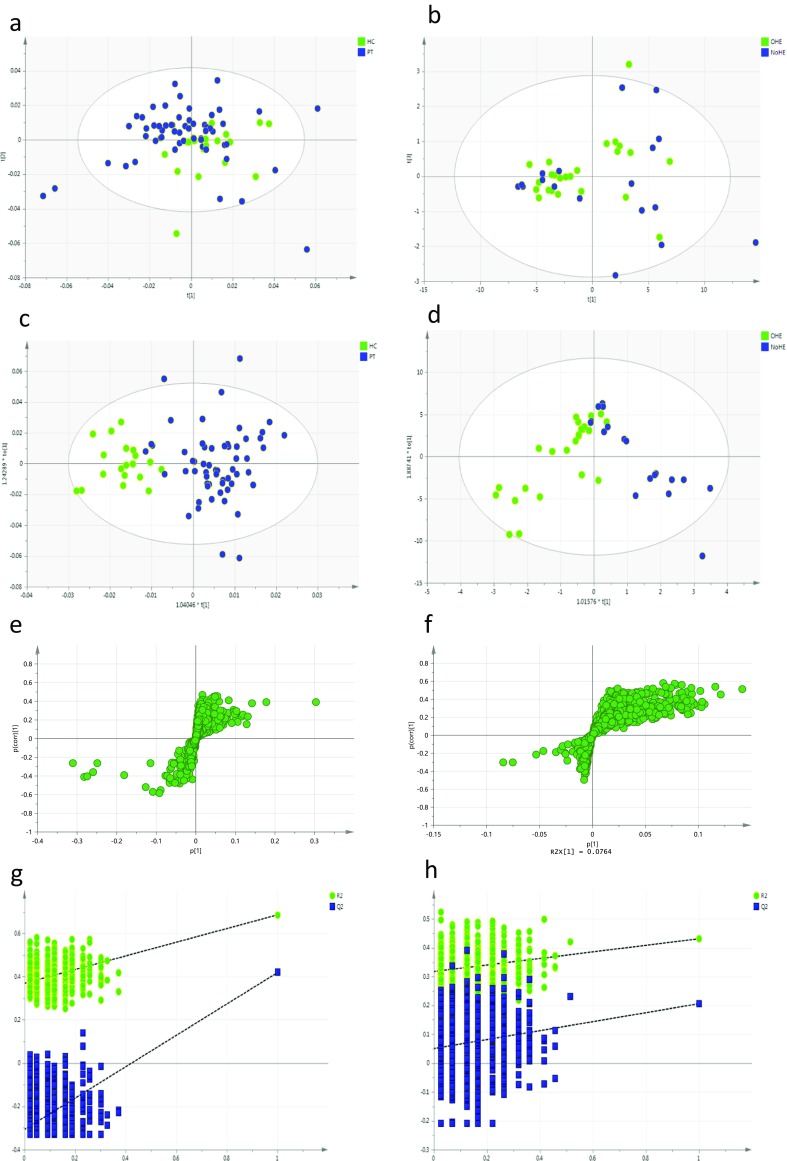
Multivariate analysis of urinary spectra comparing patients (PT) with cirrhosis and healthy controls (HC) and between patients with OHE and no HE. **a** Principal components analysis HC vs PT ((R2X = 0.674, Q2 = 0.54). **b** Principal components analysis OHE v no HE ((R2X = 0.627, Q2 = 0.463). **c** Orthogonal projection to latent squares discriminant analysis (OPLS-DA), HC v PT (R2X =0.66, R2Y = 0.479, Q2 = 0.314, 84 % and specificity of 95 %). **d** OPLS-DA OHE v no HE, (R2X =0.648, R2Y = 0.483, Q2 = 0.118, 100 % and specificity of 64 %). **e** S-loadings plot for model C. **f** S-loadings plot for model D. **g** Permutation analysis for PLSDA HC v PT. **h** Permutation analysis for OHE v no HE

Three-component OPLS-DA analysis demonstrates excellent discriminant ability (R2X =0.66, R2Y = 0.47, Q2 = 0.314). This gave a sensitivity of 84 % and specificity of 95 %, CV-ANOVA *p* = 0.0002 with a validation permutation analysis suggesting a valid model (see Fig. 3) with y axis crossing points of the permutations significantly different from those of the constructed model. The cross validated AUROC for the model for patients with cirrhosis versus healthy controls was 0.92 (95 % CI 0.87–0.97).

Univariate analysis of selected metabolites is summarised in Table 2 and confirm significant differences in alanine, acetate, glycine, hippurate, N-methyl nicotinic acid, phenylacetylglycine and 1-methonicotinamide between patients with cirrhosis and controls.

**Table 2:** The ^1^H-NMR spectral intensities of selected metabolites identified by urinary ^1^H-NMR spectroscopy for healthy controls, patients with cirrhosis and also patients with and without OHE. Data are given as mean (SD), [arbitrary units, normalised to total spectral area, log transformed where necessary], (resonances used for univariate analysis are in bold)

Peak # from Fig. 2	Metabolite	Chemical shifts ppm	Controls (*n* = 17)	Cirrhosis (*n* = 52)	*P*-value, controls vs cirrhotics	No HE (*n* = 21)	cHE (*n* = 7)	OHE (n = 17)	*P*-value, No HE vs cHE vs OHE
1	TSP	0				N.A.			
2	Lactate	**1.33 (d)**	7.26(1.84)	6.55(2.20)	0.235	6.03(1.16)	6.02(1.60)	7.13(2.74)	0.222
3	Alanine	**1.48 (d)**	7.50(2.09)	6.19(2.30)	0.041	5.42(2.04)	6.05(2.95)	6.70(2.29)	0.232
4	Acetate	**1.92 (s)**	4.87(3.59)	3.49(1.68)	0.031	3.17(0.74)	3.11(1.51)	3.98(2.17)	0.231
5	Pyruvate	**2.37 (s)**	1.98(0.27)	1.90(0.82)	0.694	2.01(1.23)	1.54(0.62)	1.97(0.51)	0.349
6	Citrate	**2.57 (d)**, 2.70 (d)	25.40(8.12)	26.80(15.5)	0.718	22.10(14.1)	29.00(12.3)	28.80(17.5)	0.357
7	Dimethylamine	**2.72 (s)**	6.01(0.15)	6.60(2.53)	0.373	7.23(1.99)	5.72(2.17)	6.19(1.85)	0.120
8	Creatinine	**3.05 (s)**, 4.05 (s)	88.77(19.2)	75.2(28.2)	0.070	82.20(35.11)	78.6(30.8)	71.3(12.1)	0.459
9	Trimethylamine-*N*-oxide	**3.27 (s)**	22.00(8.44)	25.80(3.85)	0.689	19.80(27.7)	44.8(73.3)	25.3(29)	0.306
10	Glycine	**3.56 (s)**	11.50(3.28)	8.64(3.58)	0.003	8.31(2.40)	8.05(4.84)	8.81(3.95)	0.838
11	Hippurate	**3.98 (d),** 7.56 (t), 7.65 (t), **7.84 (d)**	20.70(13.1)	7.65(1.13)	<0.001	5.02(5.75)	13.3(7.67)	7.27(11.9)	0.232
12	Formate	**8.46 (s)**	0.83(0.62)	1.26(1.15)	0.149	1.36(1.01)	1.48(1.18)	1.17(1.32)	0.762
13	Histidine*	7.76 (s)	0.46(0.06)	0.47(0.03)	0.942	0.27(0.04)	0.21(0.07)	0.68(0.06)	0.052
14	N-methyl nicotinic acid	8.84 (m), **9.13 (s)**	1.03(0.83))	0.44(0.47)	<0.001	0.72(0.60)	0.39(0.26)	0.25(0.29)	0.004
15	Glutamate	**2.04 (m)**, 2.28 (m)	7.23(0.82)	7.47(1.75)	0.589	8.01(2.14)	6.46(1.23)	7.48(1.50)	0.098
16	Acetylcarnitine*	**3.20 (s)**	4.00(1.36)	4.53(2.59)	0.560	5.33(5.76)	3.73(1.35)	4.27(1.82)	0.499
17	Phenlyacetylglycine	**7.36 (m)**, 7.42 (m)	12.23(4.68)	9.01(5.66)	0.039	6.92(4.03)	10.6(6.77)	9.87(5.98)	0.164
18	1-methylnicotinamide	8.97 (m), **9.28 (s)**	0.055(0.0066)	0.21(0.22)	0.004	0.29(0.26)	0.10(0.21)	0.20(0.16)	0.103

*Provisional assignment

Differences in the final study cohort of 45 patients with cirrhosis were then considered with respect to more detailed clinical categorisation:

## PHES-positive versus PHES-negative patients

There was no clear clustering by PHES (*n* = 16 positive, *n* = 29 negative) for the patients with cirrhosis using a three-component PCA model (R2X = 0.55, Q2 = 0.326). PLS-DA and OPLS-DA models on patients with and without positive PHES testing did not demonstrate any valid models (for example, three-component PLS-DA, R2X = 0.55, R2Y = 0.548, Q2 = −0.383) with a CV-ANOVA of 1 indicating no validity or discriminatory ability. Use of alternative scaling methods such as mean centring alone, or Pareto-scaling (with or without logarithmic transformation) did not yield valid discriminatory models.

## EEG-positive versus EEG-negative patients

PCA on the urinary ^1^H-NMR data for patients with EEG data (*n* = 23 positive, *n* = 22 negative) similarly showed no clear clustering. Further, no clear clustering was observed on unsupervised modelling for patients with or without abnormal EEG.

Supervised PLS-DA and OPLS-DA models using patients with normal or abnormal EEG did not discriminate between these groups. For example, on 3-component PLS-DA, R2X = 0.435, R2Y = 0.53, Q2 = 0.245 with a CV-ANOVA of 1 indicating no validity or discriminatory ability. This did not improve using alternative scaling methods.

## OHE versus no OHE (either cHE or no HE)

PLS-DA and OPLS-DA models using patients with OHE (*n* = 21) versus patients with either cHE or no HE (*n* = 24) did not discriminate between these groups. On PLS-DA, the R2 was 0.446 and the Q2–0.21 for these models with a CV ANOVA of 1 indicating no validity or discriminatory ability. This did not improve on the removal of weak outliers.

## OHE versus patients with cirrhosis and no HE

When comparing patients with OHE (n = 21) with patients with no HE (*n* = 17), it was possible to generate valid models to describe this difference in clinical phenotype. On PCA, patients with HE tended to have less variance in urinary metabolic phenotype as shown in the clustering on the scores plot for a three-component PCA (R2X = 0.719, Q2 = 0.103). A three-component PLS-DA model could not robustly discriminate the urinary NMR spectra of patients with OHE from patients with no HE with R2X = 0.648, R2Y = 0.483 and Q2Y = 0.118 with a sensitivity of 100 % and specificity of 64 % (Fig. 3d). The relatively low Q2Y is suggestive of an over-fitted model although the AUROC was high. The cross validated AUROC for the model for patients with and without OHE 0.86 (95%CI 0.72–0.95).

The primary metabolites responsible for these potential differences were increases in histidine and glutamate for patients with OHE and increased N-methyl nicotinic acid (in patients without HE). Taking N-methyl nicotinic acid levels alone, these predicted the presence of OHE with 80 % sensitivity and 65 % specificity, AUROC 0.722 (95 % CI 0.580–0.865, *p* = 0.002). Univariate analysis of selected metabolite intensities is provided in Table 2. In the absence of a fully validated model these markers should be tested in larger cohorts or using other measurement modalities (such as LCMS).

## cHE versus patients with cirrhosis and no HE

PLS-DA and OPLS-DA models using patients with cHE (*n* = 7) versus patients with normal PHES and EEG (*n* = 17) did not discriminate between these groups. On PLS-DA, the R2 was 0.383 and the Q2 was –0.33 for 2 component models with a CV ANOVA of 1 indicating no validity or discriminatory ability. This did not improve on the removal of weak outliers.

## Nutritional status

Utilising a nutritional scoring system (0, 1, 2) for each patient PLS-DA and OPLS-DA models using patients with any HE (OHE or cHE) did not discriminate between these groups. Only a subset of patients (*n* = 28) had a nutritional assessment performed and these were split into two groups (low or intermediate (*n* = 9) versus high risk (*n* = 19) of malnutrition). On PLS-DA the R2 was 0.378 and the Q2 was –0.15 for these models with a CV ANOVA of 1, indicating no validity or discriminatory ability.

## Discussion

Consistent with previous studies, we illustrate that urinary ^1^H-NMR metabolic profiling discriminates patients with cirrhosis from healthy controls. In addition to a reduction in hippurate, as previously shown in cirrhosis, compared to healthy controls (Bajaj 2014;Shariff et al. 2011), in this cohort we have also identified a reduction in alanine, acetate, glycine, N-methyl nicotinic acid and phenylacetylglycine in cirrhosis patients with a concomitant an increase in 1-metholnicotinamide. We have further demonstrated that urinary ^1^H-NMR metabolic profiling has the potential to detect a urinary metabolic phenotype associated with OHE (albeit with less validity), but the technique has not been of value in characterising cHE. However, in contrast to plasma NMR studies, urinary ^1^H-NMR profiling did not correlate with severity of underlying liver disease (expressed as MELD). When the differing clinical, neuropsychological or neurophysiological tests were included, this suggests that the urinary metabolic profile does not discriminate between clinical grading, even with an improved sample size over other recent studies.

We did note a change in N-methyl nicotinic acid levels in patients with cirrhosis and between those with or without OHE. Urinary N-methyl nicotinic acid excretion was impaired in patients with cirrhosis and further impaired in patents with OHE. Whether nicotinamide metabolism is altered in cirrhosis is a matter of debate (Pumpo et al. 2001) , although we have not seen reports to date on changes in metabolism and urinary levels in patients with HE. Our results favour the hypothesis of reduction in methylation secondary to cirrhosis, leading to reduced urinary N-methyl nicotinic acid secretion, in contrast to the results from Pumpo et al. (Pumpo et al. 2001). Lower urinary N-methyl nicotinic acid levels predicted the presence of OHE and these nucleotide pathways should be further evaluated. It may be that our cohort has a higher incidence of HE than that of Pumpo et al. (Pumpo et al. 2001), given that patients without HE had levels close to the normal range for healthy controls.

Other investigators have focused on the effects of medication on the metabolome and microbiome. Rifaximin has been shown to have profound effects on the serum metabolic profile in patients with cHE (Bajaj et al. 2011). Particular changes were seen in increases in both saturated and unsaturated fatty acids after rifaximin administration. These were measured by GCMS and are therefore not entirely comparable with our results here. Of note, rifaximin itself did not modulate the gut microbiome significantly, other than small changes in two species (Eubacteriaceae and Veillonellaceae). This was while highly significant changes in cognitive function were observed. In patients undergoing planned withdrawal from lactulose, cognition was linked to stool *Prevotella* spp. concentrations and to changes in choline metabolism, with TMAO being the only urinary metabolite to be different in those with HE recurrence (Bajaj et al. 2012).

The effect of gut microbial activity on cHE on the background of the changes already observed in patients with cirrhosis (Bajaj et al. 2012;Bajaj et al. 2013) may not be large, which may partly explain why we did not observe differences in the urinary metabolic profile in patients with cirrhosis with or without cHE, but those with OHE demonstrated more marked differences. Both of these previous studies had high levels of patient characterisation (psychometric evaluation, gut microbe pyrosequencing, urine and plasma ^1^H-NMR spectroscopy and in vivo ^1^H cerebral magnetic resonance spectroscopy), but had small numbers of participants, with only seven in the study of lactulose withdrawal (Bajaj et al. 2012) and 20 in the study discussing rifaximin (Bajaj et al. 2013). Our present study seems to point to cHE not being responsible for a significant shift in the urinary metabolic profile, which is likely borne out by the small effect on the gut microbiome, demonstrated in these previous studies.

In common with other top down spectroscopic techniques urinary NMR gives an overview of several other reaction pathways which may contribute to cognitive dysfunction in these groups. Experimentally, induction of zinc deficiency-related encephalopathy is possible with oral ingestion of histidine (increasing renal excretion of zinc). This may be a cofactor in the encephalopathy we see in patients with HE. It may be more likely that histidine is incorporated into the brain in the context of hyperammonaemia causing increased cerebral histamine levels (Fogel et al. 1990) and subsequent neurotransmitter abnormalities (in particular increased GABAergic tone).

It is now understood that even modest increases in circulating ammonia occur in patients with OHE and increased levels of glutamate secondary to this have been demonstrated by NMR spectroscopy in plasma of patients with acute liver failure. The kidney is a major ammonia detoxifying organ and hence by-products of this reaction are likely to be seen in the urine of patients with cirrhosis and HE (Shawcross et al. 2011).

It is unclear with methylation of nicotinic acid may be singularly affected in HE. Our metabolite coverage does not allow us to interrogate all other methylation reactions but cognitive dysfunction related to methylation has been described classically in pellagra (Pitsavas et al. 2004) and in the schizophrenias.

The patients in this cohort were predominantly ambulant and without overt sepsis or other known precipitating causes. As OHE in patients with cirrhosis is multifactorial in aetiology, studies of the serum and urinary metabolic profile of the specific effects of sepsis, gastrointestinal bleeding or electrolyte disturbance would be of interest. Septic encephalopathy is substantially different from HE and it also occurs in non-cirrhotic patients, so the mental state of cirrhosis patients whose clinical condition is complicated by sepsis is difficult to examine clearly. These patients were therefore excluded from this study. Similarly, patients with major electrolyte imbalance, with the exception of hyponatremia, which is so closely associated with encephalopathy, were excluded.

The main weakness of the study are the relatively small sample size and single modality of metabonomics platform. The validity statistics for the model of patients with OHE versus no HE suggest that further characterisation of larger validation cohorts would be needed to confirm these finding and generate valid multivariate models with more confidence in the discriminatory ability of these markers. Tandem use of mass spectrometry may be useful to better resolve the intensities of glutamine and glutamate and give wider metabolite coverage.

We did explore whether nutritional state could confound the urinary metabolic profile, but our findings demonstrated no significant change. The significant changes between healthy controls and patients with cirrhosis in terms of creatinine, creatine and glutamate suggest that changes in muscle metabolism are being reflected in the urinary metabolic profile. However, within the cohort of patients with cirrhosis, nutritional phenotype differences were not reflected in the urinary NMR profile. Further direct measurements of changes in muscle, serum and urine in the same patient cohort may be of more use to make further conclusions.

In conclusion, the present data show that metabolic profiling of urine by NMR spectroscopy is not currently of value in supporting a clearer diagnosis of covert HE in patients with cirrhosis. Whether some of the potential biomarkers of nucleotide metabolism identified in the overt HE situation could be quantified by other methods, such as mass spectrometry, requires further consideration. Examination of other matrices, such as blood plasma, saliva or stool may give further profiling information for use in the diagnosis of HE and in the understanding of underlying pathogenic mechanisms.
